# Operational Definition of Liver Cancer in Studies Using Data from the National Health Insurance Service: A Systematic Review

**DOI:** 10.15430/JCP.2023.28.2.47

**Published:** 2023-06-30

**Authors:** Yu Rim Kim, Ji Yoon Baek, Seung Hee Seo, Hyeree Park, Sooyoung Cho, Aesun Shin, sup, 6, sup

**Affiliations:** 1 College of Medicine, Ewha Womans University, Seoul, Korea; 2 Cancer Research Institute, Seoul National University, Seoul, Korea; 3 Interdisciplinary Program in Cancer Biology Major, Seoul National University College of Medicine, Seoul, Korea; 4 Integrated Major in Innovative Medical Science, Seoul National University Graduate School, Seoul, Korea; 5 Department of Preventive Medicine, Seoul National University College of Medicine, Seoul, Korea; 6 Medical Research Center, Genomic Medicine Institute, Seoul National University College of Medicine, Seoul, Korea

**Keywords:** Systematic review, Liver neoplasms, Incidence, National health programs

## Abstract

Data from the Korean National Health Insurance Service (NHIS) have been widely used to provide real-world evidence. Due to the nature of claims data, researchers use operational definitions to define patients with specific diseases. This study aimed to conduct a systematic review of the operational definitions of liver cancer used in studies based on the NHIS database and to suggest the most appropriate operational definition. Literature search was completed on January 6, 2021, using PubMed and KoreaMed. We applied the most frequently used operational definitions of liver cancer to the NHIS–National Sample Cohort and calculated age-standardized incidence rates (ASRs) of liver cancer by year. The ASRs using each operational definition were compared with the ASR from the Korea Central Cancer (KCCR) data. Among 236 articles, 90 were selected for review, covering histologically various kinds of liver cancer and varied by study subjects. Most studies (n = 79) did not mention whether the codes for the operational definition were from only the main diagnosis or from both the main and sub-diagnosis. The most frequently used operational definition was C22 (n = 39); however, the most similar operational definition was the ASR using “C22.0 or C22.9” for men and “C22.0” for women as the main diagnosis to the ASR from the KCCR. Based on the comparison with KCCR data, we suggest using “C22.0 or C22.9” for men and “C22.0” for women as the main diagnosis for the operational definition of liver cancer when using the NHIS data.

## INTRODUCTION

Liver cancer is one of the five major cancers in Korea [1]. According to the Korea Central Cancer Registry (KCCR), the incidence rate of liver cancer ranks seventh (fifth in male cancer incidence and sixth in female cancer incidence) among all cancers [2]. It is the second leading cause of cancer mortality following lung cancer [2,3]. While the incidence of liver cancer has continuously decreased, the five-year age-standardized net survival rate of liver cancer was 27.2%in 2019 [2], showing a poor prognosis. There are pathologically various forms of liver cancer, such as hepatocellular carcinoma (HCC), cholangiocarcinoma, hepatoblastoma, and angiosarcoma. HCC accounts for 70%-75% of all liver cancer incidences, and cholangiocarcinoma accounts for 12%-15% [4,5]. However, it is difficult to distinguish HCC or cholangiocarcinoma from all liver cancers when conducting epidemiological studies with National Health Insurance Service (NHIS) data because they do not include information on the pathological diagnosis.

The number of studies using real-world data, such as National Health Insurance claims data, is increasing [6]. The NHIS covers almost 97% of the entire population (3% for medical aid beneficiaries) [7]. Its data are actively used for epidemiological studies, as it contains patients’ diagnosis codes, the treatments they receive, and drug prescriptions if they receive any [8]. Previous studies that compared the NHIS database with the National Cancer Registry or with medical records from a hospital contended that NHIS can be a worthwhile resource to use for research if proper algorithms are applied [9,10]. However, due to the nature of claim data, the diagnostic codes used in the database may not accurately reflect the diagnosis of diseases [6,11-13].

For these reasons, it is essential to set an operational definition of target diseases during study design. When selecting operational definitions, the characteristics of the data source and whether it is appropriate to define a patient by using only the diagnosis code of the claim data should be considered [12]. In previous studies using the NHIS database, various operational definitions were used to define liver cancer patients [14-17]. According to a previous study, the number of cancer occurrences according to operational definitions with NHIS claim data was overestimated compared with that from KCCR data [11]. In a report published by NHIS Ilsan Hospital, the incidence rates calculated by 6 different operational definitions, such as “International Classification of Diseases, 10th Revision (ICD-10) disease code for the main diagnosis” or “ICD-10 disease code for both main and sub-diagnosis”, were overestimated compared with the actual incidence rate in 8 cancers, including liver cancer [18]. Additionally, the liver cancer incidences were quite variable by each operational definition. This suggests the possibility of bias in research depending on how the definitions were made. As this previous study did not reflect the usage of the operational definitions from published literature, the purpose of this study is to systematically review the operational definitions of liver cancer in epidemiological research based on the NHIS database and to compare the liver cancer incidence derived from each operational definition to KCCR statistics. Ultimately, we aim to suggest the optimal operational definition for liver cancer when using the NHIS database.

## MATERIALS AND METHODS

### Literature search and study selection

Previous studies of liver cancer based on the NHIS database were selected. Regarding the search query, we discussed the extent of the term “liver cancer” for this study. According to the Korean Association for the Study of the Liver [1], the Korean term “Gan-am” means ‘primary cancer in the liver including intrahepatic bile duct cancer’. However, in most of the literature, the term “liver cancer” excludes bile duct cancer, and both intrahepatic and extrahepatic bile duct cancers are generally grouped as “bile duct cancer”. In this respect, the extent of “liver cancer” used in this study is identical to the definition of liver cancer given by the Korean Association for the Study of the Liver or the C22 diagnosis of the ICD-10.

We searched the medical terms of liver cancer based on the ICD-10 diagnosis codes (Table S1) by Medical Subject Headings (MeSH) terms and combined them with the “OR” operator. We also included all MeSH terms of bile duct cancer and combined them with the “OR” operator. Then, we excluded literature that describes only extrahepatic bile duct cancer by exclusion criteria because the term “liver cancer” in this study includes intrahepatic bile duct cancer only. To include all studies based on the NHIS database, we searched the full-text literature that contains “national health insurance” or “health insurance review and assessment”. Finally, we linked the search query “Korea” with the “AND” operator. We modified the same search query to fit the format of KoreaMed to include domestic studies that are not listed on PubMed. The final search query and search date of each database are shown in Table S2. We excluded duplications of studies.

The criteria of literature selection followed guidelines published by the National Evidence-based Healthcare Collaborating Agency (NECA) [19]. For the first screening, two researchers (K.Y.R. and B.J.Y.) independently scrutinized the literature by title and abstract according to exclusion criteria agreed upon by both. After the first screening, the literature that both researchers agreed not to include in the study was excluded from full-text screening.

### Age-standardized incidence rate comparison with the KCCR

To determine which definition accurately reflects the incidence of liver cancer, age-standardized incidence rates (ASRs) for the most frequently used operational definitions used in the literature were calculated in the NHIS–National Sample Cohort (NHIS-NSC) and compared with the ASR from the KCCR. The standard population used to calculate the ASR was the mid-year Korean population of 2010. Due to the very low incidence of liver cancer cases aged under 30, we calculated truncated ASR for the population aged 30 years old and over. As the enhanced benefits coverage registry program began in 2005, the enhanced benefits coverage registry code for cancer patients (V193) was also available from 2005 on. Therefore, we calculated the ASR from 2005 to 2015. To conduct a quantitative analysis between the ASRs of the operational definitions and the ASR from the KCCR, we calculated the absolute mean difference. It can be interpreted as an estimation of how far each figure is from the KCCR data on average. The absolute mean difference was calculated with the formula below.


$$\text{Absolute mean difference}=\sum \left|\left(\text{KCCR's ASR}\right)-\left(\text{operational definition's ASR}\right)\right|/11\text{ (year)}$$

If the absolute mean difference is closer to zero, the ASR of the operational definition is similar to that of the KCCR.

### Ethics statement

The study was approved by the Institutional Review Board (IRB) of Seoul National University College of Medicine/Seoul National University Hospital (approval number: E-2111-115-1273), and the written informed consent was exempt because all analyses were conducted using publicly available data without personal identification information.

## RESULTS

### Systematic review of operational definitions

**Figure 1:**
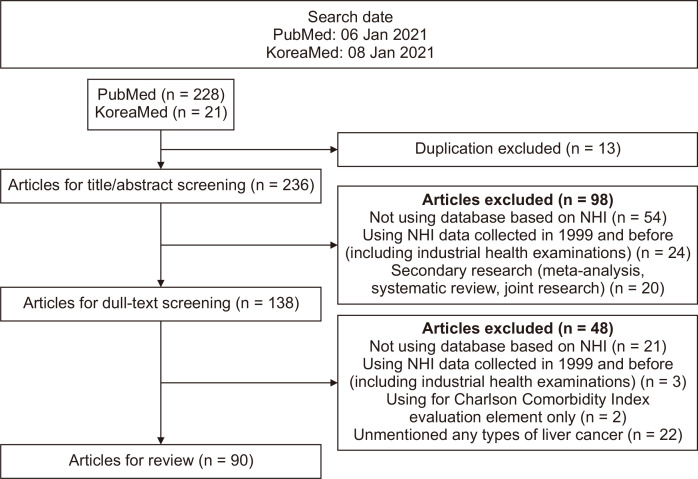
Flow chart of the study selection. NHI, National Health Insurance.

From the literature in PubMed (n = 228) and KoreaMed (n = 21), duplicates were excluded (n = 13). From 236 studies, we first excluded studies by first screening the title and abstract (n = 98) and excluded studies by screening the full text (n = 48) (Fig. 1).

There were 98 operational definitions used in the final 90 studies (Table S3). The frequency of the operational definitions used is shown in Table 1. Despite a variety of operational definitions, the common feature of every definition was the usage of ICD codes in any form. However, among 98 definitions, only 11 clearly mentioned whether the ICD code was for the main diagnosis code or both the main and sub-diagnosis.

Among the literature defining liver cancer (n = 57), the ICD-10 disease code “C22” was the most common operational definition (n = 33). In NHIS-NSC, the single code “C22” itself was assigned, but hardly any patients were assigned to it; therefore, we interpreted “C22” in the literature as “all the subcodes in ICD-10 beginning with C22” rather than “the single code C22 itself in ICD-10”. The most frequently used operational definition for HCC (n = 29) was “C22.0”, which is identical to the ICD-10 code standing for HCC (n = 8). However, the ICD code C22 includes not only HCC but also other kinds of liver cancer, such as cholangiocarcinoma or Kupffer cell sarcoma, and was still used as the operational definition for HCC (n = 5). It is the second most frequently used code after “C22.0”. Furthermore, “C22” is also used to define hepatobiliary cancer patients even though the code indicates other kinds of liver cancer.

When we analyzed 98 cases of operational definitions used with no consideration of histologic classification, the most frequently used operational definition was “C22” (n = 39). We classified the articles with the operational definition as “ICD codes” (n = 16) as a separate group from the operational definition “C22” because they do not specify which ICD codes were used. Additionally, 9 cases did not reveal how liver cancer patients were defined.

The third most commonly used operational definition with detailed code was the combination of “C22” and enhanced benefits coverage registry code “V193” (n = 5), and the combination of subcodes as “C22, C22.0, or C22.9” (n = 3) was the fourth, followed by “C22.0 or C22.9” (n = 2) and “C22.1” (n = 2). There were several other definitions as various combinations of ICD codes or combining ICD codes with specific treatment or hospitalization.

### Comparison with KCCR

We calculated the ASR per 100,000 population of operational definitions that were used more than once except those omitting “C22.0”, which indicates HCC that covers most liver cancers. We also did not calculate the ASR of “C22.0–C22.9” because it presented all the subcodes of C22 but not the single code “C22” itself and was hardly used at all; thus, it can be substituted by ASR by “C22”. Table 2 shows the ASR calculated by the 5 most frequently used operational definitions and the ASR calculated by the KCCR per 100,000 populations. As most articles did not specify whether the authors used the main diagnosis or sub-diagnosis, we calculated ASR from 5 operational definitions in both the main diagnosis and sub-diagnosis.

Table 2 shows the absolute mean differences between each ASR and KCCR for quantitative analysis. The operational definitions having the smallest mean difference from KCCR were different by sex: “C22.0 or C22.9” for men (7.00) and “C22.0” for women (3.15), followed by “C22 with V193” in both sex (7.32 for men and 3.56 for women).

**Figure 2:**
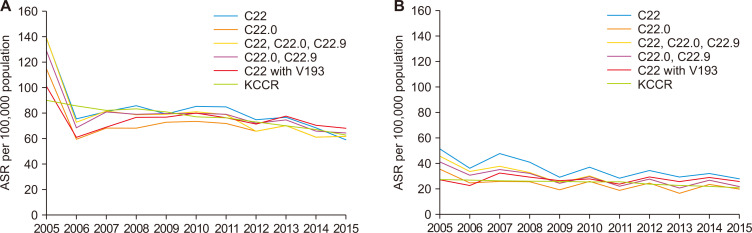
Truncated ASR (≥30 years old) per 100,000 people by the 5 most frequently used operational and KCCR. (A) Men. (B) Women. ASR, age-standardized incidence rate; KCCR, Korea Central Cancer Registry.

Figure 2 shows the ASR calculated by the 5 most frequently used operational definitions and KCCR. The top 3 operational definitions with the smallest absolute mean difference are “C22.0 or C22.9”, “C22 with V193”, and “C22, C22.0, or C22.9” for men and “C22.0”, “C22 with V193”, and “C22.0 or C22.9” for women.

## DISCUSSION

This study identified how studies define liver cancer patients using the NHIS database. Most studies defined liver cancer patients solely with the ICD-10 code, and “C22” was used most frequently. Liver cancer and intrahepatic bile duct carcinoma were mainly defined as “C22”, but HCC and hepatobiliary cancer were usually defined by subcodes of “C22”, “C22.0,” and “C22.1”, respectively. Although the most commonly used definition was “C22” (n = 39), the smallest mean difference with the KCCR was observed from “C22.0 or C22.9” (n = 2) for men and “C22.0” (n = 8) for women.

Through this study, we found some problems when defining liver cancer patients by operational definitions. Most literature (88.8%) used obscure operational definitions and did not specify whether the code was applied in the main diagnosis or sub-diagnosis or both when defining liver cancer. Among the 98 definitions used, 9 cases did not mention the operational definition they used, and 16 cases stated that the ICD-10 code was used to define liver cancer patients but did not remark on the exact combination of codes (n = 16). In 25 cases the operational definitions were not clear, which was more than three times the usage of “C22.0” (n = 8), the second most frequently used operational definition. The lack of additional information on the operational definition in the method description in studies can be regarded as a source of limited reproducibility. Additionally, most of the operational definitions comprised only ICD-10 codes, but such disease code-dependent definitions can overestimate the number of patients. As Health Insurance Review and Assessment reviews, the claim data to assess whether excessive charges were incurred in medical treatment, some diagnosis codes are assigned based on the physical examinations performed, which may not be consistent with the correct diagnosis of the disease [5,10-12]. Furthermore, treatment and medical services can be provided to patients for preventive purposes before the doctor confirms a definite diagnosis [11].

The limitation of the study was that we could not ascertain whether each patient from different operational definitions corresponded with the patients registered in the KCCR. Furthermore, the size of the sample cohort was not large enough for calculating the ASR for female liver cancer; therefore, a large annual variation in the ASR of female patients by year was observed, making it difficult to compare directly with that from the KCCR.

Even though there are other subtypes of liver cancer, such as HCC or intrahepatic bile duct carcinoma, because the KCCR only covers “liver cancer” as a whole, we could only compare each operational definition and the ASR of liver cancer from the KCCR, and it was not possible to assess other subtypes of liver cancer. Therefore, further studies are needed to compare the ASR in the KCCR calculated by each type of liver cancer, such as HCC.

The strength of this study is that we identified the optimal operational definition to apply to liver cancer-related studies through a systematic review of the literature. Additionally, this study could provide evidence of the necessity for a specific description of each operational definition when grouping patients using the NHIS database.

Based on the systematic review and comparison to the KCCR, we suggest defining liver cancer patients as “C22.0 or C22.9” for men and “C22.0” for women as the main diagnosis when using the NHIS database. Furthermore, while the results of this review suggest the optimal operational definition for patients with liver cancer, more well-designed studies with appropriate data are required to determine the optimal operational definition for HCC patients.

## Figures and Tables

**Table 1:** Frequency of operational definitions used in studies

Operational definition	Liver cancer	Hepatocellular carcinoma	Hepatobiliary cancer	Intrahepatic bile duct carcinoma	Frequency
C22	33	5	1		39
International Classification of Diseases code (no specific subcode)	8	4	4		16
No specific definition	3	3	2	1	9
C22.0		8			8
C22 with V193	3	1	1		5
C22, C22.0, C22.9		3			3
C22.0, C22.9		2			2
C22.1				2	2
C22.0–C22.9	2				2
C22.1–C22.4	2				2
C22.0, C22.1, C24			1		1
C22.0, C22.1, C22.9	1				1
C22, C22.0, C22.1	1				1
C22 and liver cancer-related death	1				1
Hospitalized with C22	1				1
Hospitalized with C22, C22.0, C22.1	1				1
3 outpatients or 1 hospitalized with C22	1				1
3 outpatients or 1 hospitalized with C22.0 or C22.9		1			1
C22.0 with surgery or other therapy		1			1
Initiation of Hepatocellular carcinoma treatment		1			1
Total	57	29	9	3	98

**Table 2:** Truncated age-standardized incidence rates (≥ 30 years old) for the 5 most frequently used operational definitions and KCCR (per 100,000)

Men												
Operational definitions	2005	2006	2007	2008	2009	2010	2011	2012	2013	2014	2015	Absolute mean difference
C22	138.6	75.5	81.1	85.8	79.2	85.3	84.9	74.8	76.7	68.4	59.0	8.56
C22.0	114.8	59.4	68.2	68.2	72.8	73.5	71.8	65.7	70.1	61.1	61.7	10.05
C22, C22.0, C22.9	139.1	72.8	81.6	78.7	79.0	81.0	78.8	72.0	74.6	65.8	64.1	7.62
C22.0, C22.9	129.0	68.5	80.9	79.0	79.7	79.8	79.1	72.0	74.6	65.8	64.4	7.00
C22 with V193	101.0	60.9	68.9	76.7	76.8	80.0	76.3	71.3	77.6	70.4	68.1	7.32
KCCR	90.0	85.7	82.1	83.3	80.9	77.0	76.3	73.0	70.0	67.1	62.7	-

Women												
Operational definitions	2005	2006	2007	2008	2009	2010	2011	2012	2013	2014	2015	Absolute mean difference
C22	51.4	36.2	47.7	41.0	29.1	37.0	28.4	34.4	29.3	32.0	27.9	11.07
C22.0	35.5	24.7	25.8	25.6	19.3	25.9	18.8	24.5	16.5	23.5	19.8	3.15
C22, C22.0, C22.9	45.7	33.7	37.7	32.8	24.7	30.2	22.1	27.7	20.7	26.8	21.8	5.87
C22.0, C22.9	41.2	30.7	35.1	32.2	24.4	29.5	22.1	27.7	20.7	26.8	21.8	4.87
C22 with V193	27.1	22.6	32.4	29.2	26.4	27.8	23.7	29.4	25.7	28.9	25.8	3.56
KCCR	27.3	26.9	26.3	26.0	25.9	25.4	25.6	23.7	22.7	22.0	20.8	-

KCCR, Korea Central Cancer Registry; -, not applicable.
