# Supplementary material for: Operational Definition of Liver Cancer in Studies Using Data from the National Health Insurance Service: A Systematic Review
Source: J Cancer Prev. 2023 Jun 30;28(2):47–52. doi: 10.15430/JCP.2023.28.2.47 (PMC10331033; doi:10.15430/JCP.2023.28.2.47)
Supplement: Supplementary file 1 [file jcp-28-2-47-supple.pdf]

Table S1. C22 in International Classification of Disease-10th revision

C22	Malignant neoplasm of liver and intrahepatic bile ducts<br>*Excl*.: Biliary tract NOS (C24.9)<br>Secondary malignant neoplasm of liver (C78.7)
C22.0	Liver cell carcinoma<br>Hepatocellular carcinoma<br>Hepatoma
C22.1	Intrahepatic bile duct carcinoma<br>Cholangiocarcinoma
C22.2	Hepatoblastoma
C22.3	Angiosarcoma of liver<br>Kuffer cell sarcoma
C22.4	Other sarcomas of liver
C22.7	Other specified carcinomas of liver
C22.9	Liver, unspecified

Table S2. Search query and search date of each database

Database	Search date	Search query
PubMed	06 Jan 2021	((liver cancer) OR (liver neoplasm) OR (Hepatic Cancer) OR (hepatic neoplasm) OR (liver tumor) OR (hepatoma) OR (malignant hepatoma) OR (liver malignant neoplasm) OR (Liver cell carcinoma) OR (Hepatocellular Cancer) OR (hepatocellular carcinoma) OR (hepatocarcinoma) OR (Intrahepatic bile duct carcinoma) OR (Intrahepatic Cholangiocarcinoma) OR (Cholangiocarcinomas) OR (Cholangiocellular Carcinoma) OR (bile duct cancer) OR (bile duct neoplasm) OR (hepatoblastoma) OR (liver AND angiosarcoma) OR (hepatic angiosarcoma) OR (Kupffer cell sarcoma) OR (C22)) AND ((national health insurance) OR (national health information database) OR (health insurance review and assessment)) AND KOREA
KoreaMed	08 Jan 2021	((““liver malignant neoplasm”[ALL] OR “Liver cell carcinoma”[ALL] OR “Cholangiocarcinomas”[ALL] OR “Cholangiocellular Carcinoma”[ALL] OR “Kupffer cell sarcoma”[ALL] OR “Intrahepatic Cholangiocarcinoma”[ALL] OR “bile duct cancer”[ALL] OR “bile duct neoplasm”[ALL] OR “hepatoblastoma”[ALL] OR “liver angiosarcoma”[ALL] OR “Intrahepatic bile duct carcinoma”[ALL] OR “hepatic angiosarcoma”[ALL] OR "liver cancer"[ALL] OR “liver neoplasm”[ALL] OR “Hepatocellular Cancer”[ALL] OR “Hepatic Cancer”[ALL] OR “hepatic neoplasm”[ALL] OR “liver tumor”[ALL] OR "hepatocellular carcinoma"[ALL] OR hepatocarcinoma[ALL] OR hepatoma[ALL] OR “malignant hepatoma”[ALL] OR “C22”[ALL])) AND (“national health insurance”[ALL] OR “health insurance review and assessment””[ALL])

Table S3. Summary table of studies included in the presented systematic review

No.	Author	Title of literature	Year	Database	Study subject	Operational definitions
1	Ahn et al.	Incidence and Mortality Rates of Second Pancreatic Cancer Among Survivors of Digestive Cancers: A Nationwide Population-based Study	2019	NHI-SSI	Liver cancer	C22.0–22.9
2	Ahn et al.	Cancer Development in Patients with COPD: A Retrospective Analysis of The National Health Insurance Service-National Sample Cohort in Korea	2020	NHI-NSC	Liver cancer	C22
3	Ahn et al.	Independent Risk Factors for Hepatocellular Carcinoma Recurrence after Direct-Acting Antiviral Therapy in Patients with Chronic Hepatitis C	2021	HIRA	Hepatocellular carcinoma	C22.0, C22.9/Initiation of hepatocellular carcinoma treatment
4	An et al.	Sex-specific Associations Between Serum Hemoglobin Levels and The Risk of Cause-specific Death in Korea Using The National Health Insurance Service-National Health Screening Cohort (NHIS HEALS)	2019	National Health Insurance Service-National Health Screening Cohort	Liver cancer	C22
5	Bae et al.	Risk Factors of Cardiovascular Disease according to Alcohol Behavioral Change after Cancer Diagnosis	2020	National Health Insurance Service Health-Examinee Cohort	Hepatobiliary	No specific definition
6	Bahk et al.	Age- and Cause-Specific Contributions to The Life Expectancy Gap between Medical Aid recipients and National Health Insurance Beneficiaries in Korea, 2008–2017	2020	NHID	Liver cancer	C22
7	Cho et al.	Statistical Methods for Elimination of Guarantee-time Bias in Cohort Studies: A Simulation Study	2017	NHID	Hepatocellular carcinoma	No specific definition
8	Cho et al.	Use of Cyclooxygenase Inhibitor and The Risk of Hepatocellular Carcinoma in Patients with Chronic Hepatitis B: A Nested Case-control Study Using A Nationwide Population-based Data	2020	NHIS-NSC	Hepatocellular carcinoma	C22.0 with surgery or other therapy
9	Choi et al.	Risk of Hepatocellular Carcinoma in Patients Treated With Entecavir vs Tenofovir for Chronic Hepatitis B: A Korean Nationwide Cohort Study	2019	NHIS	Hepatocellular carcinoma	C22
10	Choi and Park	Suicide Risk after Cancer Diagnosis among Older Adults: A Nationwide Retrospective Cohort Study	2020	NHIS–SC	Liver cancer	C22
11	Choi et al.	The Incidence and Management Trends of Metastatic Spinal Tumors in South Korea: A Nationwide Population-based Study	2020	HIRA	Liver cancer	ICD code (no specific subcode)
12	Choi et al.	Association between High Fatty Liver Index and Development of Colorectal Cancer: A Nationwide Cohort Study with 21,592,374 Korean	2020	NHIC	Liver cancer	C22
13	Chung et al.	Comorbidities and Prescribed Medications in Korean Patients with Chronic Hepatitis C: A Nationwide, Population-Based Study	2021	NHI	Hepatocellular carcinoma	C22, C22.0, C22.9/ C22.0, C22.9
14	Chung et al.	Liver Cirrhosis and Cancer: Comparison of Mortality	2018	NHIS–NSC	Liver cancer	C22
15	Han et al.	Cancer Risk in Patients with Intestinal Behçet's Disease: A Nationwide Population-based Study	2018	NHI	Liver cancer	C22
16	Heo et al.	Second Primary Cancer after Liver Transplantation in Hepatocellular Carcinoma: A Nationwide Population-Based Study	2017	HIRA	Hepatocellular carcinoma	C22.0
17	Heo et al.	Psychiatric Comorbidities among Patients Undergoing Liver Transplantation in South Korea: A Nationwide Population-based Study	2018	HIRA	Hepatocellular carcinoma	No specific definition
18	Hong et al.	Incidence of Extrahepatic Cancers among Individuals with Chronic Hepatitis B or C Virus Infection: A Nationwide Cohort Study	2020	NHI-NSC	Liver cancer	3 outpatient or 1 hospitalization with C22

Table S3. Continued

No.	Author	Title of literature	Year	Database	Study subject	Operational definitions
19	Hong et al.	Risk of Disease Transformation and Second Primary Solid Tumors in Patients with Myeloproliferative Neoplasms	2019	NHIS	Liver cancer	ICD code (no specific subcode)
20	Hong et al.	Bone Metastasis and Skeletal-related Events in Patients with Solid Cancer: A Korean Nationwide Health Insurance Database Study	2020	NHI-NSC	Liver cancer	C22
21	Hong et al.	Long-term Survival of 10,116 Korean Live Liver Donors	2021	NHI-NSC	Hepatobiliary	No specific definition
22	Hong et al.	Trends and Patterns of Hepatocellular Carcinoma Treatment in Korea	2016	HIRA	Hepatocellular carcinoma	C22.0
23	Huang et al.	Gallstones, Cholecystectomy and The Risk of Hepatobiliary and Pancreatic Cancer: A Nationwide Population-based Cohort Study in Korea	2020	NHI-NSC	Liver cancer/ Liver cancer/ Intrahepatic bile duct carcinoma	C22, C22.0, C22.1/ Hospitalized with C22 C22.0, C22.1/ No specific definition
24	Hwang et al.	Associations of General Obesity and Central Obesity with The Risk of Hepatocellular Carcinoma in a Korean Population: A National Population-Based Cohort Study	2021	NHIS	Hepatocellular carcinoma	C22.0
25	Hwang et al.	Diagnostic Accuracy of Administrative Database for Bile Duct Cancer by ICD-10 Code in a Tertiary Institute in Korea	2020	NHIS	Intrahepatic bile duct carcinoma/ Hepatocellular carcinoma	C22.1/C22 with V193
26	Hwangbo et al.	Incidence of Diabetes after Cancer Development: A Korean National Cohort Study	2018	NHI-NSC	Liver cancer	ICD code (no specific subcode)
27	Jang and Chang	Socioeconomic Status and Survival Outcomes in Elderly Cancer Patients: A National Health Insurance Service-Elderly Sample Cohort Study	2019	NHI-ESC	Liver cancer	C22
28	Ju et al.	Long term outcome of antiviral therapy in patients with hepatitis B associated decompensated cirrhosis	2018	HIRA	Hepatocellular carcinoma	ICD code (no specific subcode)
29	Jung et al.	Cancer Risk in Korean Patients with Behçet's Disease: A Nationwide Population-based Study	2017	NHI	Liver cancer	C22
30	Jung et al.	Cancer Risk in The Early Stages of Inflammatory Bowel Disease in Korean Patients: A Nationwide Population-based Study	2017	NHI	Liver cancer	C22
31	Kang et al.	The Clinical Utilization of Radiation Therapy in Korea between 2009 and 2013	2016	HIRA	Liver cancer	C22
32	Ki et al.	Healthcare Costs for Chronic Hepatitis C in South Korea from 2009 to 2013: An Analysis of the National Health Insurance Claims' Data	2017	HIRA	Hepatocellular carcinoma	C22 C22.0 C22.9
33	Ki et al.	Incidence, Prevalence and Complications of Budd-Chiari Syndrome in South Korea: A Nationwide, Population-based Study	2016	HIRA, RIDs	Liver cancer	ICD code for all diagnosis
34	Kim et al.	Population-based Prevalence, Incidence, and Disease Burden of Autoimmune Hepatitis in South Korea	2017	NHI, RIDs	Hepatocellular carcinoma	ICD code for all diagnosis
35	Kim et al.	Incidence and Overall Survival of Biliary Tract Cancers in South Korea from 2006 to 2015: Using the National Health Information Database	2019	NHID of the NHIS	Intrahepatic	C22.1
36	Kim et al.	Clinical Utilization of Radiation Therapy in Korea, 2016	2020	HIRA	Liver cancer	ICD code (no specific subcode)
37	Kim et al.	Effect of Statin on Hepatocellular Carcinoma in Patients with Type 2 Diabetes: A Nationwide Nested Case-control Study	2017	NHI-NSC	Hepatocellular carcinoma/Liver cancer	C22.0/C22.1–C22.4
38	Kim et al.	Statin Use And The Risk Of Hepatocellular Carcinoma In Patients At High Risk: A Nationwide Nested Case-control Study	2018	NHIS-PHEC	Hepatocellular carcinoma	C22.0

Table S3. Continued

No.	Author	Title of literature	Year	Database	Study subject	Operational definitions
39	Kim et al.	Effect of Statin Use on Liver Cancer Mortality Considering Hypercholesterolemia and Obesity in Patients with Non-Cirrhotic Chronic Hepatitis B	2019	NHIS of Korea	Liver cancer	C22 and liver cancer related death
40	Kim et al.	Incidence and Prognosis of Subsequent Cholangiocarcinoma in Patients with Hepatic Resection for Bile Duct Stones	2018	NHI-SSI	Liver cancer	C22.0–22.9
41	Kim et al.	The Association between Cancer Incidence and Family Income: Analysis of Korean National Health Insurance Cancer Registration Data	2012	Korean National Health Insurance Cancer Registration	Liver cancer	C22
42	Kim et al.	Association of Weight Change in Young Adulthood with Subsequent Risk of Hepatocellular Carcinoma: A National Cohort Study	2021	NHIS	Hepatocellular carcinoma	C22.0/No specific definition
43	Kim et al.	Association of Fasting Serum Glucose Level and Type 2 Diabetes with Hepatocellular Carcinoma in Men with Chronic Hepatitis B Infection: A Large Cohort Study	2018	NHIS	Hepatocellular carcinoma	C22.0
44	Kim et al.	Population-based Epidemiology of Primary Biliary Cirrhosis in South Korea	2016	HIRA	Hepatocellular carcinoma	ICD code for all diagnosis
45	Kim et al.	Herpes Zoster and Subsequent Cancer Risk: A Nationwide Population-based Cohort Study in Korea	2021	KNHI	Liver cancer	No specific definition
46	Kim et al.	Alcohol Consumption and Mortality From All-cause and Cancers among 1.34 Million Koreans: The Results from the Korea National Health Insurance Corporation's Health Examinee Cohort in 2000	2010	KNHIC	Liver cancer	C22
47	Kim et al.	Site-specific Cancer Risk in Patients with Type 2 Diabetes: A Nationwide Population-based Cohort Study in Korea	2020	KNHIS	Liver cancer	ICD code (no specific subcode) for primary
48	Kim et al.	Epidemiology of Intracranial Metastases in Korea: A National Cohort Investigation	2018	NHI-NSC	Liver cancer	C22
49	Kim et al.	Glomerular Hyperfiltration and Cancer: A Nationwide Population-based Study	2020	NHIS and HIRA	Liver cancer	ICD code (no specific subcode)
50	Kim et al.	Socioeconomic Burden of Cancer in Korea from 2011 to 2015	2020	NHIS	Liver cancer	C22
51	Kim et al.	Effect of Helicobacter pylori Treatment on Long-term Mortality in Patients with Hypertension	2020	NHI-NSC	Liver cancer	C22
52	Kim et al.	High Prevalence of Breast Cancer in Light Polluted Areas in Urban and Rural Regions of South Korea: An Ecologic Study on The Treatment Prevalence of Female Cancers based on National Health Insurance Data	2015	Department of Information Management of the Korean National Health Insurance Corporation	Liver cancer	C22
53	Ko et al.	Metabolic Risk Profile and Cancer in Korean Men and Women	2016	KNHIS National Sample Cohort	Liver cancer	C22
54	Kwak et al.	Differential Risk of Incident Cancer in Patients with Heart Failure: A Nationwide Population-based Cohort Study	2021	KNHIS	Hepatobiliary	C22 with V193
55	Kwon et al.	The Impact of National Surveillance for Liver Cancer: Results from Real-world Setting in Korea	2020	NHIS claims data linked with the National Liver Cancer Surveillance Program	Liver cancer	C22 with V193
56	Kwon et al.	The Incidences and Characteristics of Various Cancers in Patients on Dialysis: A Korean Nationwide Study	2019	Korean National Health Insurance, National Health Information	Hepatobiliary	C22

Table S3. Continued

No.	Author	Title of literature	Year	Database	Study subject	Operational definitions
57	Lee et al.	Incidence and Risk Factors for Psychiatric Comorbidity among People Newly Diagnosed with Cancer Based on Korean National Registry Data	2015	NHIS	Liver cancer	C22
58	Lee et al.	Cost-effectiveness of Antiviral Prophylaxis during Pregnancy for the Prevention of Perinatal Hepatitis B Infection in South Korea	2018	National Health Insurance Claims Database	Hepatocellular carcinoma	ICD code (no specific subcode)
59	Lee et al.	High level of Medication Adherence is Required to Lower Mortality in Patients with Chronic Hepatitis B Taking Entecavir: A Nationwide Cohort Study	2021	NHIS	Hepatocellular carcinoma	C22
60	Lee et al.	Association between Alzheimer's Disease and Cancer Risk in South Korea: An 11-year Nationwide Population-based Study	2018	NHIS–SC	Hepatobiliary	ICD code for all diagnosis
61	Lee et al.	The Economic Burden of Cancer Attributable to Obesity in Korea: A Population-based Cohort Study	2019	Health check-up cohort data of NHIS	Liver cancer	No specific definition
62	Lee et al.	Cancer Risk in 892,089 Patients with Psoriasis in Korea: A nationwide Population-based Cohort Study	2019	NHIS	Liver cancer	C22
63	Lee et al.	Incident Hepatocellular Carcinoma Risk in Patients Treated with a Sulfonylurea: A Nationwide, Nested, Case-control Study	2019	NHIS-NSC	Hepatocellular carcinoma/ Liver cancer	C22.0/C22.1–C22.4
64	Lee et al.	Economic Burden of Cancer in Korea during 2000–2010	2015	National Health Insurance Claims Data	Liver cancer	C22
65	Lee et al.	Socioeconomic Costs of Liver Disease in Korea	2011	National Health Insurance Statistical Yearbook	Liver cancer	C22
66	Lee et al.	Cancer Risk in Road Transportation Workers: A National Representative Cohort Study with 600,000 Person-years of Follow-up	2020	KNHIS	Liver cancer	C22
67	Lee et al.	Association of Metabolic Risk Factors With Risks of Cancer and All-Cause Mortality in Patients With Chronic Hepatitis B	2021	KNHIS	Hepatocellular carcinoma	C22
68	Myung et al.	Cancer Incidence according to The National Health Information Database in Korean Patients with End-stage Renal Disease Receiving Hemodialysis	2020	NHID	Liver cancer	C22 with V193
69	Nam et al.	Obesity Fact Sheet in Korea, 2018: Data Focusing on Waist Circumference and Obesity-related Comorbidities	2019	KNHIS Health Examination Database	Liver cancer	C22 with V193
70	Nguyen et al.	The Economic Burden of Cancers Attributable to Smoking in Korea, 2014	2019	NHIS	Liver cancer	C22
71	Oh et al.	Incidence risk of various types of digestive cancers in patients with pre-dialytic chronic kidney disease: A nationwide population-based cohort study	2018	NHIS-NSC	Hepatobiliary	C22.0, C22.1, C24
72	Oh et al.	Long-Term Glucocorticoid Use and Cancer Risk: A Population-Based Cohort Study in South Korea	2020	NHIS	Liver cancer	C22
73	Oh et al.	Cancer Risk in Korean Patients with Gout	2022	Korean NIH	Liver cancer	C22
74	Park et al.	The Prevalence of Depression among Patients with the Top Ten Most Common Cancers in South Korea	2017	Health Insurance Review and Assessment Service-National Patient Sample	Liver cancer	C22
75	Park et al.	Cancer Risk in Patients with Parkinson's Disease in South Korea: A Nationwide, Population-based Cohort Study	2019	HIRA	Liver cancer	C22
76	Park et al.	Job Loss and Re-employment of Cancer Patients in Korean Employees: A Nationwide Retrospective Cohort Study	2008	NHI	Liver cancer	No specific definition

Table S3. Continued

No.	Author	Title of literature	Year	Database	Study subject	Operational definitions
77	Park and Song	Medical Care Costs of Cancer in The Last Year of Life Using National Health Insurance Data in Korea	2018	HIRA	Liver cancer	C22
78	Seo et al.	The Clinical Utilization of Radiation Therapy in Korea between 2011 and 2015	2018	HIRA	Liver cancer	C22
79	Shim et al.	Reduced Liver Cancer Mortality with Regular Clinic Follow-up among Patients with Chronic Hepatitis B: A Nationwide Cohort Study	2020	NHIS	Liver cancer	C22.0, C22.1, C22.9
80	Shim et al.	Serum Alanine Aminotransferase Level and Liver-related Mortality in Patients with Chronic Hepatitis B: A Large National Cohort Study	2018	NHIS	Liver cancer	C22
81	Sinn et al.	Late Presentation of Hepatitis B among Patients with Newly Diagnosed Hepatocellular Carcinoma: A National Cohort Study	2019	NHI-NSC	Hepatocellular carcinoma	3 outpatient or 1 hospitalization with C22.0 C22.9
82	Sohn et al.	A Nationwide Study of Surgery in a Newly Diagnosed Spine Metastasis Population	2019	HIRA	Hepatobiliary	ICD code (no specific subcode)
83	Sohn et al.	Nationwide Epidemiology and Healthcare Utilization of Spine Tumor Patients in The Adult Korean Population, 2009–2012	2015	HIRA	Hepatobiliary	ICD code (no specific subcode)
84	Sohn et al.	A Nationwide Epidemiological Study of Newly Diagnosed Spine Metastasis in The Adult Korean Population	2016	HIRA	Hepatobiliary	ICD code (no specific subcode)
85	Suh et al.	Prediction of Future Hepatocellular Carcinoma Incidence in Moderate to Heavy Alcohol Drinkers with the FIB-4 Liver Fibrosis Index	2015	Korean National Health Insurance Medical Service Claims Data	Hepatocellular carcinoma	C22
86	Suh et al.	Risk Factors for Developing Liver Cancer in People with and without Liver Disease	2018	NHIS-NSC	Liver cancer	C22 with hospitalization
87	Sung et al.	Temporal Relationship between Idiopathic Inflammatory Myopathies and Malignancies and Its Mortality: A Nationwide Population-based Study	2020	KNHIS	Liver cancer	ICD code (no specific subcode)
88	Yang et al.	Cost of Chronic Hepatitis B Infection in South Korea	2004	National Health Insurance Corporation Database, NHI Database	Hepatocellular carcinoma	C22
89	Yang et al.	The Societal Burden of HBV-related Disease: South Korea	2010	HIRA	Hepatocellular carcinoma	C22, C22.0, C22.9
90	You et al.	Metformin and Gastrointestinal Cancer Development in Newly Diagnosed Type 2 Diabetes: A Population-based Study in Korea	2020	KNHIS	Liver cancer	C22

ICD: International Classification of Disease; NHI, National Health Insurance; NHI-SSI, National Health Insurance-Support for Serious Illness; NHI-NSC, National Health Insurance Service-National Sample Cohort; HIRA, Health Insurance Review and Assessment Service; NHIS, National Health Insurance Service; NHID, National Health Information Database; NHIS–SC, National Health Insurance Service–Senior Cohort; NHIS–NSC, National Health Insurance Service–National Sample Cohort; NHI-ESC, National Health Insurance Service-Elderly Sample Cohort; NHIS-PHEC, National Health Insurance Service Physical Health Examination Cohort; KNHIC, Korea National Health Insurance Corporation; KNHIS, Korean National Health Insurance Service; RIDs: Rare intractable diseases.

REFERENCES

1. Ahn HS, Kang TU, Swan H, Kang MJ, Kim N, Kim HJ, et al. Incidence and Mortality Rates of Second Pancreatic Cancer Among Survivors of Digestive Cancers: A Nationwide Population-Based Study. Pancreas 2019;48:412-419.
2. Ahn SV, Lee E, Park B, Jung JH, Park JE, Sheen SS, et al. Cancer development in patients with COPD: a retrospective analysis of the National Health Insurance Service-National Sample Cohort in Korea. BMC Pulm Med 2020;20:170.
3. Ahn YH, Lee H, Kim DY, Lee HW, Yu SJ, Cho YY, et al. Independent Risk Factors for Hepatocellular Carcinoma Recurrence after Direct-Acting Antiviral Therapy in Patients with Chronic Hepatitis C. Gut Liver 2021;15:410-419.
4. An Y, Jang J, Lee S, Moon S, Park SK. Sex-specific Associations Between Serum Hemoglobin Levels and the Risk of Cause-specific Death in Korea Using the National Health Insurance Service-National Health Screening Cohort (NHIS HEALS). J Prev Med Public Health 2019;52:393-404.
5. Bae EM, Cho IY, Jun JH, Lee K, Kim JY, Bae WK, et al. Risk Factors of Cardiovascular Disease according to Alcohol Behavioral Change after Cancer Diagnosis. Korean J Fam Med 2020;41:222-228.
6. Bahk J, Kang HY, Khang YH. Age- and cause-specific contributions to the life expectancy gap between Medical Aid recipients and National Health Insurance beneficiaries in Korea, 2008-2017. PLoS One 2020;15:e0241755.
7. Cho IS, Chae YR, Kim JH, Yoo HR, Jang SY, Kim GR, et al. Statistical methods for elimination of guarantee-time bias in cohort studies: a simulation study. BMC Med Res Methodol 2017;17:126.
8. Cho KS, Sohn W, Lee YC, Chi SA, Cho JY, Kim K, et al. Use of cyclooxygenase inhibitor and the risk of hepatocellular carcinoma in patients with chronic hepatitis B: A nested case-control study using a nationwide population-based data. J Viral Hepat 2020;27:68-73.
9. Choi J, Kim HJ, Lee J, Cho S, Ko MJ, Lim YS. Risk of Hepatocellular Carcinoma in Patients Treated With Entecavir vs Tenofovir for Chronic Hepatitis B: A Korean Nationwide Cohort Study. JAMA Oncol 2019;5:30-36.
10. Choi JW, Park EC. Suicide risk after cancer diagnosis among older adults: A nationwide retrospective cohort study. J Geriatr Oncol 2020;11:814-819.
11. Choi SH, Koo JW, Choe D, Kang CN. The Incidence and Management Trends of Metastatic Spinal Tumors in South Korea: A Nationwide Population-based Study. Spine (Phila Pa 1976) 2020;45:E856-e863.
12. Choi YJ, Lee DH, Han KD. Association between high fatty liver index and development of colorectal cancer: a nationwide cohort study with 21,592,374 Korean. Korean J Intern Med 2020;35:1354-1363.
13. Chung JW, Choi HY, Ki M, Jang ES, Jeong SH. Comorbidities and Prescribed Medications in Korean Patients with Chronic Hepatitis C: A Nationwide, Population-Based Study. Gut Liver 2021;15:295-306.
14. Chung W, Jo C, Chung WJ, Kim DJ. Liver cirrhosis and cancer: comparison of mortality. Hepatol Int 2018;12:269-276.
15. Han M, Jung YS, Kim WH, Cheon JH, Park S. Cancer Risk in Patients with Intestinal Behçet's Disease: A Nationwide Population-Based Study. Gut Liver 2018;12:433-439.
16. Heo J, Noh OK, Oh YT, Chun M, Kim L. Second primary cancer after liver transplantation in hepatocellular carcinoma: a nationwide population-based study. Hepatol Int 2017;11:523-528.
17. Heo J, Noh OK, Oh YT, Chun M, Kim L. Psychiatric comorbidities among patients undergoing liver transplantation in South Korea: a nationwide population-based study. Hepatol Int 2018;12:174-180.
18. Hong CY, Sinn DH, Kang D, Paik SW, Guallar E, Cho J, et al. Incidence of extrahepatic cancers among individuals with chronic hepatitis B or C virus infection: A nationwide cohort study. J Viral Hepat 2020;27:896-903.
19. Hong J, Lee JH, Byun JM, Lee JY, Koh Y, Shin DY, et al. Risk of disease transformation and second primary solid tumors in patients with myeloproliferative neoplasms. Blood Adv 2019;3:3700-3708.
20. Hong S, Youk T, Lee SJ, Kim KM, Vajdic CM. Bone metastasis and skeletal-related events in patients with solid cancer: A Korean nationwide health insurance database study. PLoS One 2020;15:e0234927.
21. Hong SK, Choe S, Yi NJ, Shin A, Choe EK, Yoon KC, et al. Long-term Survival of 10,116 Korean Live Liver Donors. Ann Surg 2021;274:375-382.
22. Hong YM, Yoon KT, Cho M, Kang DH, Kim HW, Choi CW, et al. Trends and Patterns of Hepatocellular Carcinoma Treatment in Korea. J Korean Med Sci 2016;31:403-409.
23. Huang D, Lee J, Song N, Cho S, Choe S, Shin A. Gallstones, Cholecystectomy and the Risk of Hepatobiliary and Pancreatic Cancer: A Nationwide Population-based Cohort Study in Korea. J Cancer Prev 2020;25:164-172.
24. Hwang S, Park YM, Han KD, Yun JS, Ko SH, Ahn YB, et al. Associations of general obesity and central obesity with the risk of hepatocellular carcinoma in a Korean population: A national population-based cohort study. Int J Cancer 2021;148:1144-1154.
25. Hwang YJ, Park SM, Ahn S, Lee J, Park YS, Kim N. Diagnostic accuracy of administrative database for bile duct cancer by ICD-10 code in a tertiary institute in Korea. Hepatobiliary Pancreat Dis Int 2020;19:575-580.
26. Hwangbo Y, Kang D, Kang M, Kim S, Lee EK, Kim YA, et al. Incidence of Diabetes After Cancer Development: A Korean National Cohort Study. JAMA Oncol 2018;4:1099-1105.
27. Jang BS, Chang JH. Socioeconomic status and survival outcomes in elderly cancer patients: A national health insurance service-elderly sample cohort study. Cancer Med 2019;8:3604-3613.
28. Ju YC, Jun DW, Choi J, Saeed WK, Lee HY, Oh HW. Long term outcome of antiviral therapy in patients with hepatitis B associated

decompensated cirrhosis. World J Gastroenterol 2018;24:4606-4614.
29. Jung YS, Han M, Kim DY, Cheon JH, Park S. Cancer risk in Korean patients with Behçet's disease: A nationwide population-based study. PLoS One 2017;12:e0190182.
30. Jung YS, Han M, Park S, Kim WH, Cheon JH. Cancer Risk in the Early Stages of Inflammatory Bowel Disease in Korean Patients: A Nationwide Population-based Study. J Crohns Colitis 2017;11:954-962.
31. Kang JK, Kim MS, Jang WI, Seo YS, Kim HJ, Cho CK, et al. The clinical utilization of radiation therapy in Korea between 2009 and 2013. Radiat Oncol J 2016;34:88-95.
32. Ki M, Choi HY, Kim KA, Jang ES, Jeong SH. Healthcare Costs for Chronic Hepatitis C in South Korea from 2009 to 2013: An Analysis of the National Health Insurance Claims' Data. Gut Liver 2017;11:835-842.
33. Ki M, Choi HY, Kim KA, Kim BH, Jang ES, Jeong SH. Incidence, prevalence and complications of Budd-Chiari syndrome in South Korea: a nationwide, population-based study. Liver Int 2016;36:1067-1073.
34. Kim BH, Choi HY, Ki M, Kim KA, Jang ES, Jeong SH. Population-based prevalence, incidence, and disease burden of autoimmune hepatitis in South Korea. PLoS One 2017;12:e0182391.
35. Kim BW, Oh CM, Choi HY, Park JW, Cho H, Ki M. Incidence and Overall Survival of Biliary Tract Cancers in South Korea from 2006 to 2015: Using the National Health Information Database. Gut Liver 2019;13:104-113.
36. Kim E, Jang WI, Kim MS, Paik EK, Kim HJ, Yoo HJ, et al. Clinical utilization of radiation therapy in Korea, 2016. J Radiat Res 2020;61:249-256.
37. Kim G, Jang SY, Han E, Lee YH, Park SY, Nam CM, et al. Effect of statin on hepatocellular carcinoma in patients with type 2 diabetes: A nationwide nested case-control study. Int J Cancer 2017;140:798-806.
38. Kim G, Jang SY, Nam CM, Kang ES. Statin use and the risk of hepatocellular carcinoma in patients at high risk: A nationwide nested case-control study. J Hepatol 2018;68:476-484.
39. Kim GA, Shim JJ, Lee JS, Kim BH, Kim JW, Oh CH, et al. Effect of Statin Use on Liver Cancer Mortality Considering Hypercholesterolemia and Obesity in Patients with Non-Cirrhotic Chronic Hepatitis B. Yonsei Med J 2019;60:1203-1208.
40. Kim HJ, Kang TU, Swan H, Kang MJ, Kim N, Ahn HS, et al. Incidence and Prognosis of Subsequent Cholangiocarcinoma in Patients with Hepatic Resection for Bile Duct Stones. Dig Dis Sci 2018;63:3465-3473.
41. Kim JM, Kim HM, Jung BY, Park EC, Cho WH, Lee SG. The association between cancer incidence and family income: analysis of Korean National Health Insurance cancer registration data. Asian Pac J Cancer Prev 2012;13:1371-1376.
42. Kim K, Choi S, Lee G, Son JS, Kim KH, Park SM. Association of weight change in young adulthood with subsequent risk of hepatocellular carcinoma: a national cohort study. Eur J Cancer Prev 2021;30:211-219.
43. Kim K, Choi S, Park SM. Association of fasting serum glucose level and type 2 diabetes with hepatocellular carcinoma in men with chronic hepatitis B infection: A large cohort study. Eur J Cancer 2018;102:103-113.
44. Kim KA, Ki M, Choi HY, Kim BH, Jang ES, Jeong SH. Population-based epidemiology of primary biliary cirrhosis in South Korea. Aliment Pharmacol Ther 2016;43:154-162.
45. Kim M, Han K, Yoo SA, Lee JH. Herpes Zoster and Subsequent Cancer Risk: A Nationwide Population-Based Cohort Study in Korea. Dermatology 2021;237:73-78.
46. Kim MK, Ko MJ, Han JT. Alcohol consumption and mortality from all-cause and cancers among 1.34 million Koreans: the results from the Korea national health insurance corporation's health examinee cohort in 2000. Cancer Causes Control 2010;21:2295-2302.
47. Kim SK, Jang JY, Kim DL, Rhyu YA, Lee SE, Ko SH, et al. Site-specific cancer risk in patients with type 2 diabetes: a nationwide population-based cohort study in Korea. Korean J Intern Med 2020;35:641-651.
48. Kim T, Song C, Han JH, Kim IA, Kim YJ, Kim SH, et al. Epidemiology of Intracranial Metastases in Korea: A National Cohort Investigation. Cancer Res Treat 2018;50:164-174.
49. Kim Y, Lee S, Lee Y, Kang MW, Park S, Park S, et al. Glomerular Hyperfiltration and Cancer: A Nationwide Population-Based Study. Cancer Epidemiol Biomarkers Prev 2020;29:2070-2077.
50. Kim YA, Lee YR, Park J, Oh IH, Kim H, Yoon SJ, et al. Socioeconomic Burden of Cancer in Korea from 2011 to 2015. Cancer Res Treat 2020;52:896-906.
51. Kim YI, Kim YA, Lee JW, Kim HJ, Kim SH, Kim SG, et al. Effect of Helicobacter pylori Treatment on Long-term Mortality in Patients with Hypertension. Gut Liver 2020;14:47-56.
52. Kim YJ, Lee E, Lee HS, Kim M, Park MS. High prevalence of breast cancer in light polluted areas in urban and rural regions of South Korea: An ecologic study on the treatment prevalence of female cancers based on National Health Insurance data. Chronobiol Int 2015;32:657-667.
53. Ko S, Yoon SJ, Kim D, Kim AR, Kim EJ, Seo HY. Metabolic Risk Profile and Cancer in Korean Men and Women. J Prev Med Public Health 2016;49:143-152.
54. Kwak S, Kwon S, Lee SY, Yang S, Lee HJ, Lee H, et al. Differential risk of incident cancer in patients with heart failure: A nationwide population-based cohort study. J Cardiol 2021;77:231-238.
55. Kwon JW, Tchoe HJ, Lee J, Suh JK, Lee JH, Shin S. The Impact of National Surveillance for Liver Cancer: Results from Real-World Setting in Korea. Gut Liver 2020;14:108-116.
56. Kwon SK, Han JH, Kim HY, Kang G, Kang M, Kim YJ, et al. The Incidences and Characteristics of Various Cancers in Patients on Dialysis: a Korean Nationwide Study. J Korean Med Sci 2019;34:e176.

57. Lee BO, Choi WJ, Sung NY, Lee SK, Lee CG, Kang JI. Incidence and risk factors for psychiatric comorbidity among people newly diagnosed with cancer based on Korean national registry data. Psychooncology 2015;24:1808-1814.
58. Lee D, Shin HY, Park SM. Cost-effectiveness of antiviral prophylaxis during pregnancy for the prevention of perinatal hepatitis B infection in South Korea. Cost Eff Resour Alloc 2018;16:6.
59. Lee J, Cho S, Kim HJ, Lee H, Ko MJ, Lim YS. High level of medication adherence is required to lower mortality in patients with chronic hepatitis B taking entecavir: A nationwide cohort study. J Viral Hepat 2021;28:353-363.
60. Lee JE, Kim D, Lee JH. Association between Alzheimer's Disease and Cancer Risk in South Korea: an 11-year Nationwide Population-Based Study. Dement Neurocogn Disord 2018;17:137-147.
61. Lee JE, Nam CM, Lee SG, Park S, Kim TH, Park EC. The economic burden of cancer attributable to obesity in Korea: A population-based cohort study. Eur J Cancer Care (Engl) 2019;28:e13084.
62. Lee JH, Kim HJ, Han KD, Kim HN, Park YM, Lee JY, et al. Cancer risk in 892 089 patients with psoriasis in Korea: A nationwide population-based cohort study. J Dermatol 2019;46:95-102.
63. Lee JY, Jang SY, Nam CM, Kang ES. Incident Hepatocellular Carcinoma Risk in Patients Treated with a Sulfonylurea: A Nationwide, Nested, Case-Control Study. Sci Rep 2019;9:8532.
64. Lee KS, Chang HS, Lee SM, Park EC. Economic Burden of Cancer in Korea during 2000-2010. Cancer Res Treat 2015;47:387-398.
65. Lee S, Chung W, Hyun KR. Socioeconomic costs of liver disease in Korea. Korean J Hepatol 2011;17:274-291.
66. Lee W, Kang MY, Kim J, Lim SS, Yoon JH. Cancer risk in road transportation workers: a national representative cohort study with 600,000 person-years of follow-up. Sci Rep 2020;10:11331.
67. Lee YB, Moon H, Lee JH, Cho EJ, Yu SJ, Kim YJ, et al. Association of Metabolic Risk Factors With Risks of Cancer and All-Cause Mortality in Patients With Chronic Hepatitis B. Hepatology 2021;73:2266-2277.
68. Myung J, Choi JH, Yi JH, Kim I. Cancer incidence according to the National Health Information Database in Korean patients with end-stage renal disease receiving hemodialysis. Korean J Intern Med 2020;35:1210-1219.
69. Nam GE, Kim YH, Han K, Jung JH, Park YG, Lee KW, et al. Obesity Fact Sheet in Korea, 2018: Data Focusing on Waist Circumference and Obesity-Related Comorbidities. J Obes Metab Syndr 2019;28:236-245.
70. Nguyen TXT, Han M, Oh JK. The economic burden of cancers attributable to smoking in Korea, 2014. Tob Induc Dis 2019;17:15.
71. Oh HJ, Lee HA, Moon CM, Ryu DR. Incidence risk of various types of digestive cancers in patients with pre-dialytic chronic kidney disease: A nationwide population-based cohort study. PLoS One 2018;13:e0207756.
72. Oh TK, Song IA. Long-Term Glucocorticoid Use and Cancer Risk: A Population-Based Cohort Study in South Korea. Cancer Prev Res (Phila) 2020;13:1017-1026.
73. Oh YJ, Lee YJ, Lee E, Park B, Kwon JW, Heo J, et al. Cancer risk in Korean patients with gout. Korean J Intern Med 2022;37:460-467.
74. Park B, Youn S, Yi KK, Lee SY, Lee JS, Chung S. The Prevalence of Depression among Patients with the Top Ten Most Common Cancers in South Korea. Psychiatry Investig 2017;14:618-625.
75. Park JH, Kim DH, Park YG, Kwon DY, Choi M, Jung JH, et al. Cancer risk in patients with Parkinson's disease in South Korea: A nationwide, population-based cohort study. Eur J Cancer 2019;117:5-13.
76. Park JH, Park EC, Park JH, Kim SG, Lee SY. Job loss and re-employment of cancer patients in Korean employees: a nationwide retrospective cohort study. J Clin Oncol 2008;26:1302-1309.
77. Park M, Song I. Medical care costs of cancer in the last year of life using national health insurance data in Korea. PLoS One 2018;13:e0197891.
78. Seo YS, Kim MS, Kang JK, Jang WI, Kim HJ, Cho CK, et al. The Clinical Utilization of Radiation Therapy in Korea between 2011 and 2015. Cancer Res Treat 2018;50:345-355.
79. Shim JJ, Kim GA, Oh CH, Kim JW, Myung J, Kim BH, et al. Reduced liver cancer mortality with regular clinic follow-up among patients with chronic hepatitis B: A nationwide cohort study. Cancer Med 2020;9:7781-7791.
80. Shim JJ, Kim JW, Oh CH, Lee YR, Lee JS, Park SY, et al. Serum alanine aminotransferase level and liver-related mortality in patients with chronic hepatitis B: A large national cohort study. Liver Int 2018;38:1751-1759.
81. Sinn DH, Kang D, Kang M, Paik SW, Guallar E, Cho J, et al. Late presentation of hepatitis B among patients with newly diagnosed hepatocellular carcinoma: a national cohort study. BMC Cancer 2019;19:286.
82. Sohn S, Chung CK, Han KD, Jung JH, Hyeun JH, Kim J, et al. A Nationwide Study of Surgery in a Newly Diagnosed Spine Metastasis Population. J Korean Neurosurg Soc 2019;62:46-52.
83. Sohn S, Kim J, Chung CK, Lee NR, Park E, Chang UK, et al. Nationwide epidemiology and healthcare utilization of spine tumor patients in the adult Korean population, 2009-2012. Neurooncol Pract 2015;2:93-100.
84. Sohn S, Kim J, Chung CK, Lee NR, Park E, Chang UK, et al. A nationwide epidemiological study of newly diagnosed spine metastasis in the adult Korean population. Spine J 2016;16:937-945.
85. Suh B, Yun JM, Park S, Shin DW, Lee TH, Yang HK, et al. Prediction of future hepatocellular carcinoma incidence in moderate to heavy alcohol drinkers with the FIB-4 liver fibrosis index. Cancer 2015;121:3818-3825.
86. Suh JK, Lee J, Lee JH, Shin S, Tchoe HJ, Kwon JW. Risk factors for developing liver cancer in people with and without liver disease. PLoS One 2018;13:e0206374.
87. Sung YK, Jung SY, Kim H, Choi S, Im SG, Cha EJ, et al. Temporal relationship between idiopathic inflammatory myopathies and

malignancies and its mortality: a nationwide population-based study. Clin Rheumatol 2020;39:3409-3416.
88. Yang BM, Kim CH, Kim JY. Cost of chronic hepatitis B infection in South Korea. J Clin Gastroenterol 2004;38:S153-157.
89. Yang BM, Kim DJ, Byun KS, Kim HS, Park JW, Shin S. The societal burden of HBV-related disease: South Korea. Dig Dis Sci 2010;55:784-793.
90. You JH, Song SO, Kang MJ, Cho YY, Kim SW, Suh SH, et al. Metformin and Gastrointestinal Cancer Development in Newly Diagnosed Type 2 Diabetes: A Population-Based Study in Korea. Clin Transl Gastroenterol 2020;11:e00254.
